# γH2AX expression in cytological specimens as a biomarker of response to radiotherapy in solid malignancies

**DOI:** 10.1002/dc.23396

**Published:** 2015-12-15

**Authors:** Ketan Shah, Ramon A. Boghozian, Christiana Kartsonaki, Ketan A. Shah, Katherine A. Vallis

**Affiliations:** ^1^ Churchill HospitalOxford Cancer Centre, Oxford University Hospitals NHS TrustOxfordUnited Kingdom; ^2^ Cancer Research UK and Medical Research Council Oxford Institute for Radiation Oncology, University of OxfordOxfordUnited Kingdom; ^3^ Nuffield Department of Population HealthUniversity of OxfordOxfordUnited Kingdom; ^4^ Department of Cellular PathologyOxford University Hospitals NHS Trust, John Radcliffe HospitalOxfordUnited Kingdom

**Keywords:** γH2AX, fine‐needle aspiration cytology, biomarker, treatment monitoring

## Abstract

Many anticancer treatments, including radiotherapy, act by damaging DNA and hindering cell function and proliferation. H2AX is a histone protein directly associated with DNA that is phosphorylated to produce γH2AX that accumulates in foci in an early response to DNA double‐strand breaks, the most deleterious lesion caused by anticancer therapy. This study reports a γH2AX detection assay that has the potential to be used as a biomarker of response to guide cancer treatment. γH2AX immunostaining was applied to tumour cell specimens obtained using fine needle aspiration (FNA). Liquid‐based cytology and direct smear cytology methods were evaluated and immunostaining protocols established using FNA samples from five cancer patients. The assay was then applied to three patients before and after radiotherapy. Results demonstrate induction of γH2AX foci following treatment, persisting for as long as one week after therapy. Immunostaining for γH2AX has been successfully applied to FNA samples, providing an opportunity to evaluate γH2AX as a treatment response marker in cancer. Diagn. Cytopathol. 2016;44:141–146. © 2015 The Authors Diagnostic Cytopathology Published by Wiley Periodicals, Inc.

The histone variant, H2AX, is a highly conserved globular protein which is structurally involved in packaging and organising DNA into chromatin. It is estimated that between 2% and 25% of human cell H2A protein consists of the H2AX variant, depending on cell type.^1^ Upon phosphorylation of serine‐139 near the C‐terminus end, γH2AX rapidly localises to sites of double‐strand breaks (DSBs). As such, γH2AX is generally considered as a surrogate marker of DNA damage. Moreover, it serves as a docking site for several downstream DNA repair proteins.^2^ However, γH2AX foci are removed during DSB repair following dephosphorylation by protein phosphatase PP2A.^2^ Many anticancer treatments target the DNA of cancer cells, thereby preventing their progression and viability.^3^ Therefore repeated analysis of γH2AX expression during radiotherapy treatment may provide useful information regarding the effects of that treatment, which could be used to predict outcome, modify therapy, and monitor tumour progression. Given the potential utility of γH2AX as a response marker,^4^ the central aim of this study was to develop a method to assess γH2AX expression longitudinally during cancer treatment. 53BP1, a chromatin‐damage binding‐protein that participates in the DNA damage response (DDR) was also measured in one case to confirm that γH2AX foci as detected using the current technique, represented DNA DSBs. This study was set up under appropriate clinical research governance with a primary objective to establish a reliable method for the detection of DNA DSBs by immunocytochemistry in human tumour cells.

## Case Reports

Nine patients were recruited to the study. Fine needle aspirate (FNA) samples from the first three participants were used for liquid‐based cytology (LBC) and immunostaining method development. For the next two study subjects the methodology was modified, and direct smear cytology was used. This provided a greater number of cells per sample and more specific immunostaining. A mouse monoclonal anti‐γH2AX antibody (clone JBW301, Merck Millipore, Watford, UK) was diluted 1:800 in blocking buffer (2% bovine serum albumin in PBS‐T) and paired with an Alexa Fluor 488 conjugated goat anti‐mouse secondary antibody (Invitrogen, Paisley, UK), diluted 1:250. A rabbit polyclonal anti‐53BP1 antibody (Cell Signaling Technology, Hichin, UK; diluted 1:1000) was paired with an Alexa Fluor 594 conjugated goat anti‐rabbit secondary antibody, diluted 1:250. Both antibodies demonstrated a focal intranuclear staining pattern in human peripheral blood mononuclear cells irradiated *ex vivo* (data not shown). There was no evidence of cross‐reactivity between anti‐rabbit secondary and the mouse anti‐γH2AX primary or between anti‐mouse secondary and rabbit anti‐53BP1 primary. There was no evidence of autofluorescence in the absence of secondary antibodies. Nuclear counterstaining was achieved using Vectorshield mounting medium (Vector Laboratories, Peterborough, UK) containing 4',6‐diamidino‐2‐phenylindole (DAPI).

The remainder of the study was restricted to participants commencing anticancer therapy to enable FNA samples to be taken before and after the first treatment. One patient withdrew from the study prior to sample acquisition leaving three patients for evaluation (designated DSB07, DSB08, DSB09). To ensure reproducibility and minimise intra‐observer variability, samples were obtained using three separate passes into different parts of the tumour for each time point before and after radiotherapy treatment. The first patient, DSB07, underwent a course of radical radiotherapy to a cervical lymph node recurrence of a squamous cell carcinoma of the parapharyngeal space. FNA samples were taken immediately before and 20 minutes after the first 2 Gy fraction of radiotherapy to the area of recurrence. The samples taken after radiotherapy showed a marked increase in the number of intranuclear foci, when compared with those taken before treatment (Fig. C‐1). Given the clinical context, these were interpreted as γH2AX foci associated with radiation‐induced DNA DSBs.

**Figure 1:**
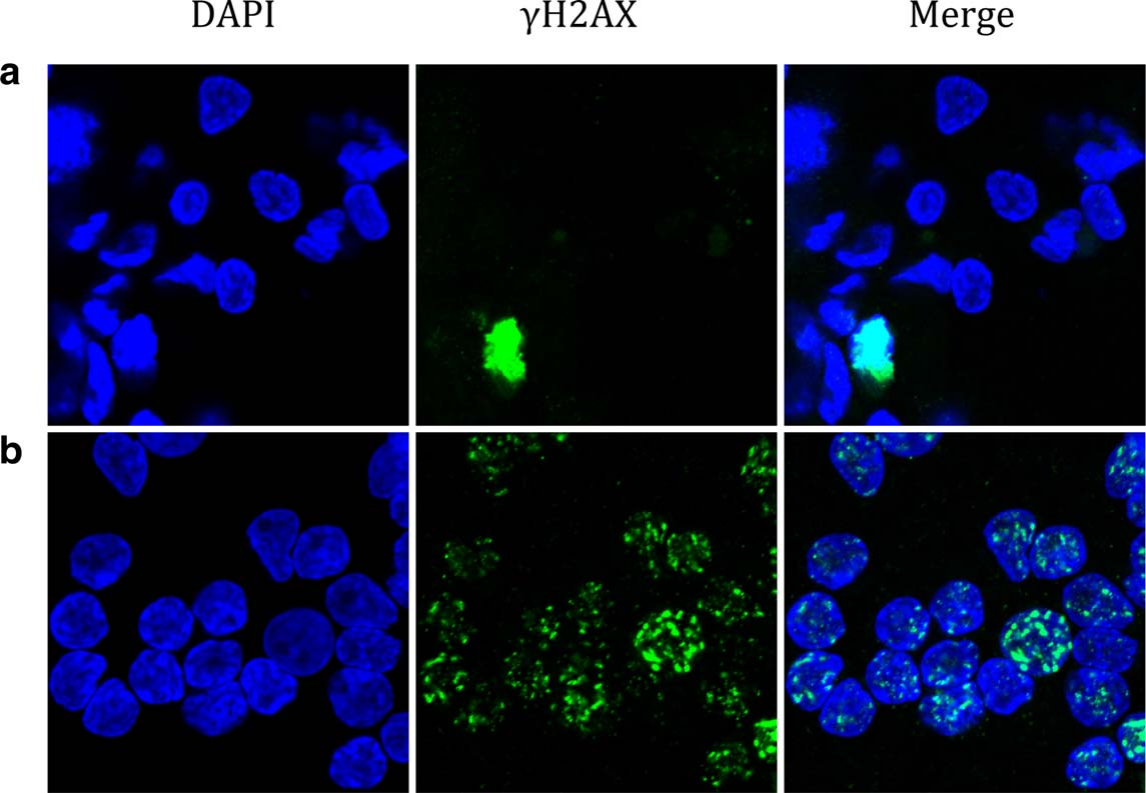
Immunocytochemistry of fine‐needle aspiration tumour specimens from metastatic squamous cell carcinoma of participant DSB07 before (a) and 20 minutes after irradiation (b) showing nucleus (DAPI, blue) and γH2AX (green). Bright green staining of single cell in (a) may represent apoptosis.

Confocal microscopy images from participant DSB07 were used to optimise image recognition software^5^ (TRI2) to automatically identify and quantify γH2AX foci within nuclear areas defined by the DAPI signal. Results of this quantification in the three samples taken before and after irradiation are shown in Figure C‐2. There was a significant increase in the number of foci following irradiation (*P* < 0.0001).

**Figure 2:**
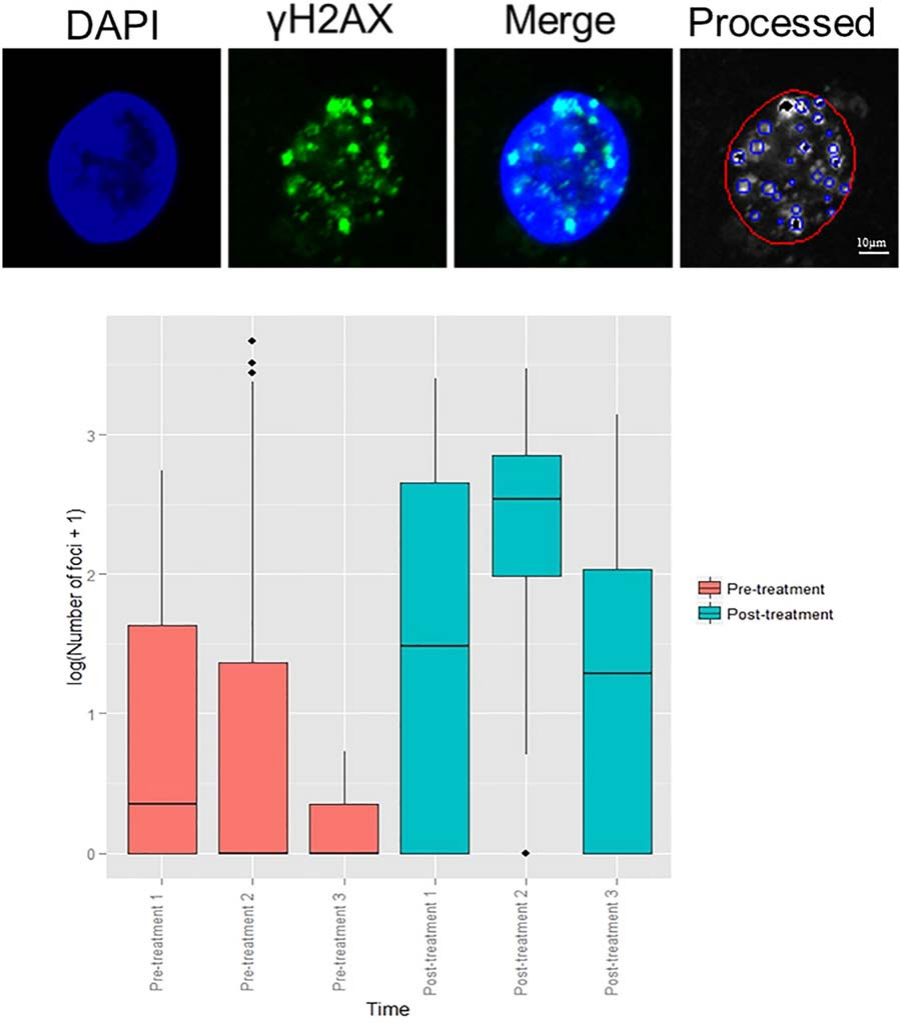
Example of automated image analysis by TRI2 software for participant DSB07. Boxplot demonstrating foci distribution in individual FNAs from participant DSB07 taken before (coral pink) and 20 minutes after (turquoise) 2 Gy of radiotherapy. *P* < 0.001 with 95% CI of (1.23, 1.48).

Participant DSB08 had a long history of non‐Hodgkins lymphoma and received palliative radiotherapy to a malignant lesion in the posterior triangle of the neck. Three FNA samples were collected immediately before and three immediately after a 3 Gy fraction of radiotherapy. Synchronous staining for 53BP1 was introduced as it has been demonstrated to co‐localise with γH2AX.^6^ The number and intensity of γH2AX foci increased with irradiation (Fig. C‐3).

**Figure 3:**
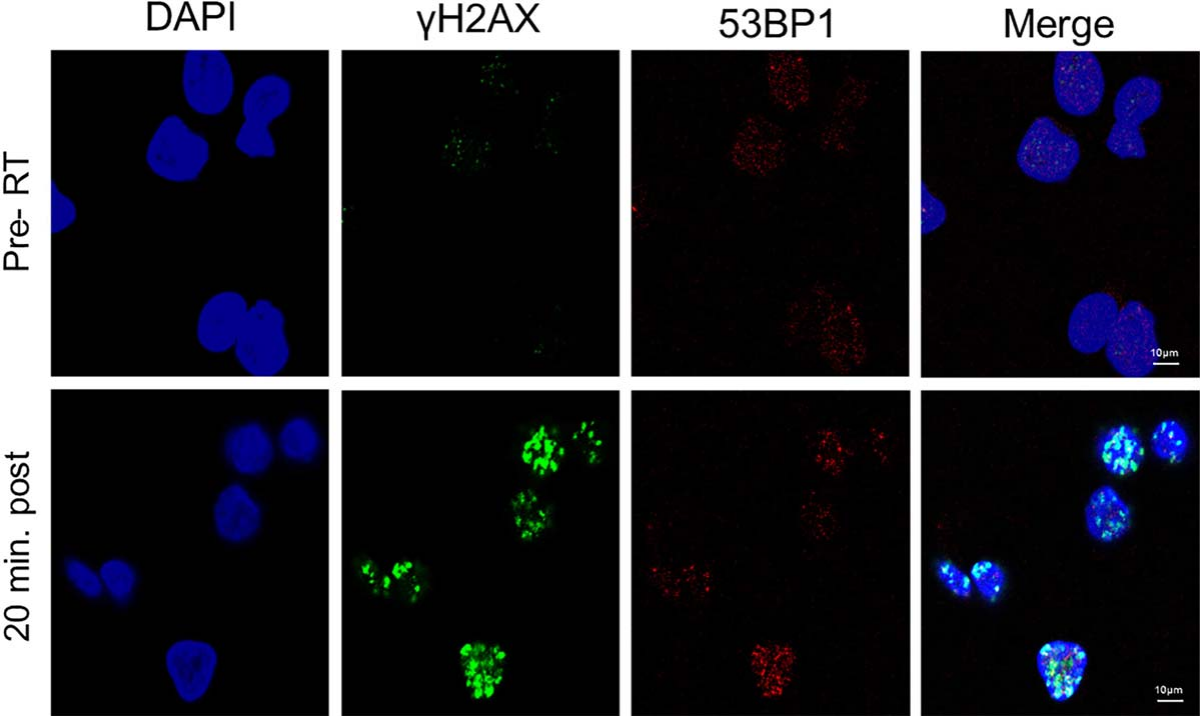
Immunocytochemistry of fine‐needle aspiration tumour specimens of non‐Hodgkins lymphoma deposits from participant DSB08 before (Pre‐RT) and 20 minutes after irradiation (20 min post‐RT) showing nucleus (DAPI, blue), γH2AX (green) and 53BP1 (red).

There was marked co‐localization of 53BP1 with γH2AX foci, before and after irradiation, however, background staining was subjectively greater than for γH2AX, making foci identification difficult. Consistent results for automated 53BP1 foci recognition could not be achieved. Baseline γH2AX foci numbers were lower for participant DSB08 compared to participant DSB07, and post‐treatment foci more numerous (Fig. C‐4).

**Figure 4:**
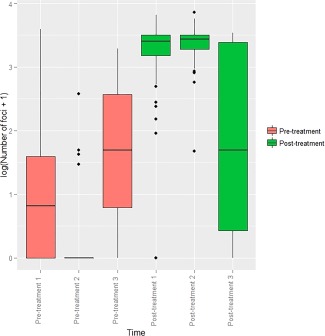
Boxplot demonstrating number of foci calculated by TRI2 image recognition in individual FNAs from non‐Hodgkins lymphoma deposits from participant DSB08 taken before (coral pink) and 20 minutes after (green) 3 Gy of radiotherapy. *P* < 0.001 with 95% CI of (1.81, 2.25).

Participant DSB09 had skin metastasis from non‐small cell lung cancer on the right upper arm. This was to be irradiated with 17 Gy divided in two fractions delivered one week apart. Samples were taken immediately before the first fraction (8.5 Gy), 20 minutes after this, and one week later, before the second fraction was delivered. The results (Fig. C‐5) demonstrate more foci before treatment than for other samples, and a very large rise in numbers of γH2AX but not 53BP1 foci 20 minutes after treatment, and most surprisingly only slightly fewer γH2AX foci one week later, largely co‐localising with 53BP1 staining.

**Figure 5:**
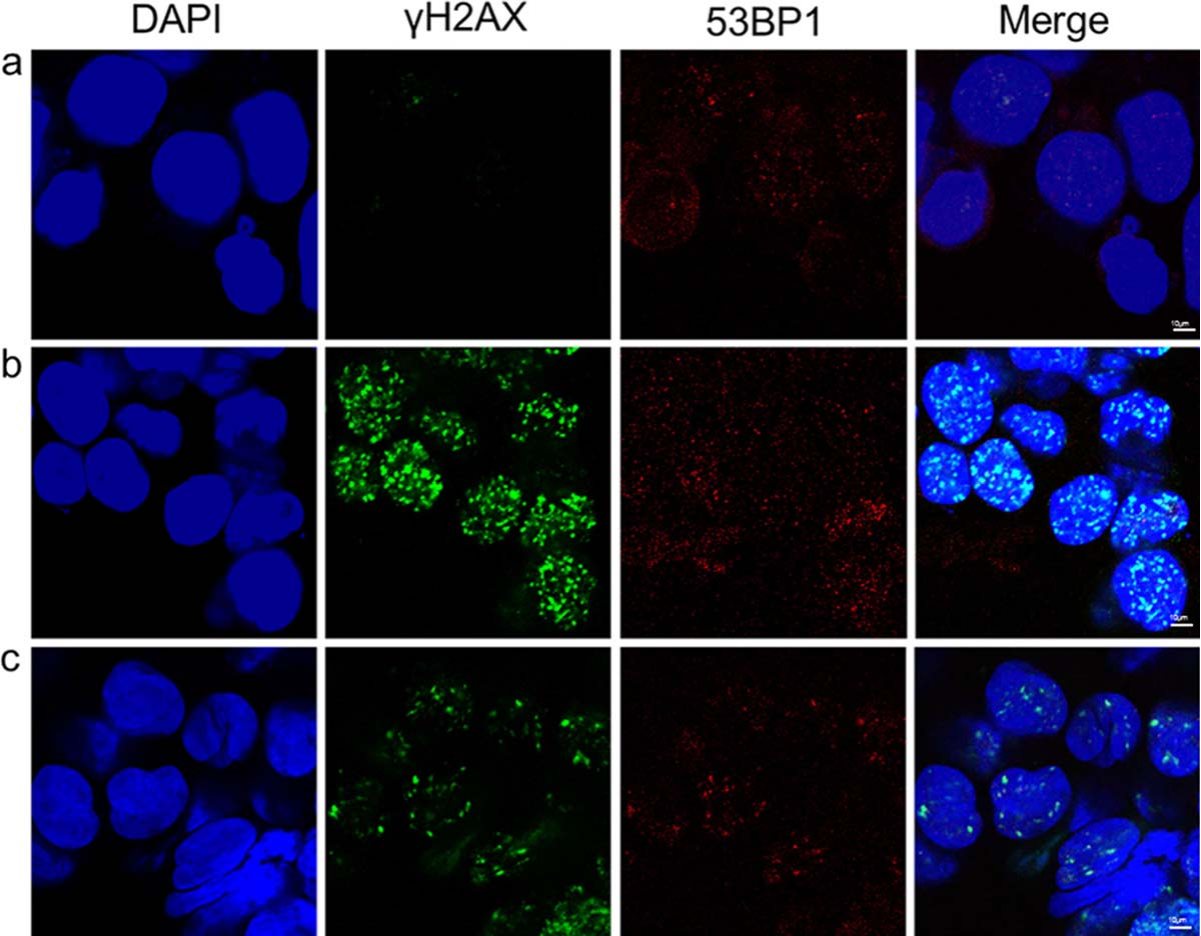
Immunocytochemistry of fine‐needle aspiration tumour specimens from metastatic non‐small cell lung cancer from participant DSB09 (a) before, (b) 20 minutes after, and (c) 1 week after irradiation. Nucleus (DAPI, blue), γH2AX (green), 53BP1 (red) and merged image.

Automated foci quantification was performed using TR12 software for all FNA samples corresponding to DSB09 participant (Fig. C‐6).

**Figure 6:**
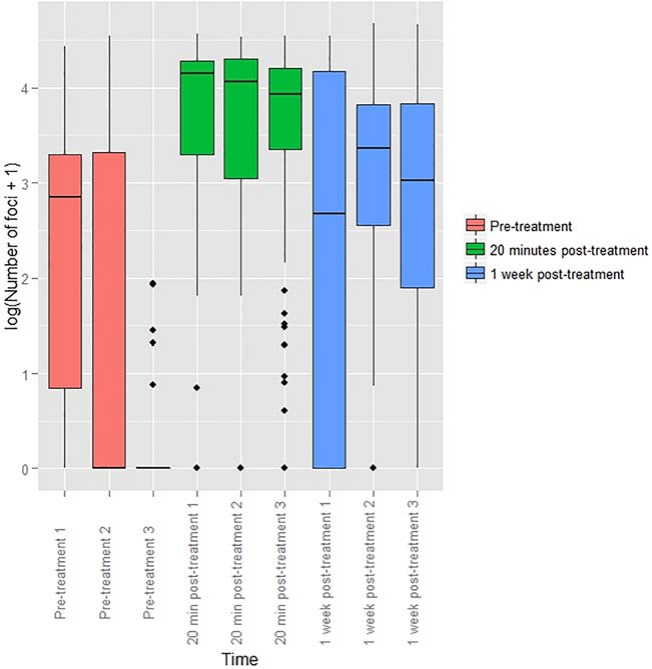
Boxplot demonstrating number of foci calculated by TRI2 image recognition in individual FNAs from metastatic nonsmall cell lung cancer from participant DSB09 taken before (coral pink), 20 minutes after (green) and 1 week after (blue) 8.5 Gy of radiotherapy. *P* < 0.001 with 95% CI of (−2.24, −1.47) for pre‐treatment vs. 20 min post‐treatment. *P* < 0.001 with 95% CI of (−0.90, −0.38) for 20 min post vs. 1 week later.

Intraclass correlation coefficients (ICC) for the transformed number of foci for each patient were calculated to show how much of the variability was because of actual differences before and after treatment rather than measurement error. They were all greater than 0.7, suggesting that in this study most of the variability was between time points. However, ICC values may be limited in their interpretation, given the small sample size of this current study.

## Discussion

This study was designed to assess the feasibility of γH2AX quantification in tumours before and after radiation treatment using FNA cytology. We also sought to evaluate the reproducibility of the assay under a variety of conditions.

The ability to track DNA damage in a tumour following genotoxic treatment may provide valuable insight into a patient's response to therapy. Data from the literature have demonstrated that tracking DNA damage using radioimmunoconjugates to target γH2AX is in fact possible.^7^ Extending this concept to the use of γH2AX immunostaining using FNA sampling, presents another tool for assessment of anticancer treatment response. There are several potential advantages for using this clinical assay. With serial sampling, physicians may be able to better assess dynamic changes and adjust treatment schedules accordingly. Furthermore, gathering FNA samples is relatively simple and tolerated by most patients with accessible tumours.

Samples from two participants were used to develop a LBC method for the γH2AX immunocytochemical assay. LBC is useful for the standardisation and preservation of cell quality during sample processing. Commercial LBC sample processing platforms developed for cervical cytology are routinely applied to different cytological specimen types including FNA,^8^ but lack the flexibility for novel assay development. In particular, cell fixation is restricted under processor guarantee conditions to proprietary solutions, the composition of which is not in the public domain. The direct smear method was therefore favoured for methodological development in this study.

Samples from participant DSB07 were obtained before and after a 2 Gy fraction of radiotherapy. They provided the first indication of the success of the assay in identifying an increase in γH2AX signal in a focal staining pattern. The magnitude of this increase was quantified using automated image recognition. Setting the parameters of the image recognition algorithm does depend on the subjective judgement of the operator, in the same way that manual foci counting is subjective. However, the automated method then provides for rapid, standardised foci counting. This approach has been adopted by other researchers seeking to translate a γH2AX foci assay for possible clinical applications.

The persistence of γH2AX foci observed in samples from the study subject with skin metastasis (DSB09) was a particularly interesting finding since there is contrary data to suggest that the majority of ionizing radiation (IR) induced γH2AX foci resolve rapidly following repair.^9^ However, there is evidence to suggest that there are two types of foci, including those which are transient and those which persist over a longer duration of time. The persistence of foci in this case may be representative of slow ongoing repair or unrepairable damage. Since γH2AX foci do not always correlate directly to DNA DSBs,^4^ co‐staining with 53BP1 was included to add more information about the nature of the foci that were demonstrated. Further data using this marker would help interpretation of the assay.

There are few published data from tumour samples in cancer patients with which to compare these results for γH2AX expression. One study reported γH2AX immunohistochemistry in 18 sets of formalin‐fixed paraffin‐embedded cervical biopsies before and 24 hours after 1.8–2.0 Gy fractions of radiotherapy.^10^ Foci numbers reported appeared to be lower than in the current study. The proportion of cells with any γH2AX foci was presented, ranging from 8–20% at baseline to 0–62% after treatment. The apparently higher pretreatment foci numbers in these results could be because of any of the differences in tumour, timing, and methodology that exist between the two studies. However it may be that the FNA assay is more sensitive than tissue immunohistochemistry.

There are reports of cytological γH2AX assay, using circulating tumour cells (CTCs).^11^, ^12^, ^13^ In one study, a threshold in the γH2AX FACS signal was used to categorise cells as positive or negative. The proportion of positive cells increased from 0–7% to 22–64% with chemotherapy treatment, and a mean of only 38 cells were examined.^11^ The extent to which FNA or CTC represents the tumour as a whole is uncertain, although FNA can potentially sample across a whole tumour mass, rather than merely cells that are able to enter the circulation. Either cytological assay involves a small sample of tumour cells. The relationship of a cytological DNA damage assay to overall tumour response‐related clinical outcomes could only be assessed empirically. More reproducible results may be obtained with the use of a threshold for “positive” cells. However, information about the distribution of foci numbers is lost.

The current study considers the role of γH2AX assays as a general marker of treatment‐induced DNA damage. Further data on assay variability using a larger number of samples would be needed in order to judge the assay's potential for application to clinical questions, including informing sample size calculations. γH2AX immunostaining of FNA samples could then be considered as a response biomarker in studies of new radiotherapy or cytotoxic treatment protocols.
